# Supplementary material for: 125Te and 57Fe nuclear resonance vibrational spectroscopic characterization of intermediate spin state mixed-valent dimers
Source: Nat Commun. 2025 Jul 25;16:6843. doi: 10.1038/s41467-025-62118-w (PMC12297287; doi:10.1038/s41467-025-62118-w)
Supplement: Supplementary file 1 — Supplementary Information [file 41467_2025_62118_MOESM1_ESM.pdf]

Supplemental Information

for

^{125}Te and ^{57}Fe Nuclear Resonance Vibrational Spectroscopic Characterization of Intermediate Spin State Mixed-Valent Dimers

Aleksa Radović[1], Justin T. Henthorn[1,§], Hongxin Wang[2], Deepak Prajapat[3], Ilya Sergeev[3], Nobumoto Nagasawa[4], Yoshitaka Yoda[4], Stephen P. Cramer[2], George E. Cutsail III[1,5,6,*]

[1]Max Planck Institute for Chemical Energy Conversion, Stiftstr. 34-36, D-45470 Mülheim an der Ruhr, Germany. [2]SETI Institute Mountain View, California 94043, United States. [3]Deutsches Elektronen-Synchrotron DESY, Notkestr. 85, D-22607 Hamburg, Germany. [4]Precision Spectroscopy Division, SPring-8/JASRI, Sayo, Hyogo 679-5198, Japan. [5]Institute of Inorganic Chemistry, University of Duisburg-Essen, Universitätsstr. 5-7, D-45141, Germany. [6]Department of Chemistry, Ludwig-Maximilians-Universität München, Butenandstr. 5-13, D-81377 Munich, Germany. [§]Present Address: School of Chemistry, University College Dublin, Belfield, Dublin 04, Ireland.

* george.cutsail@cec.mpg.de

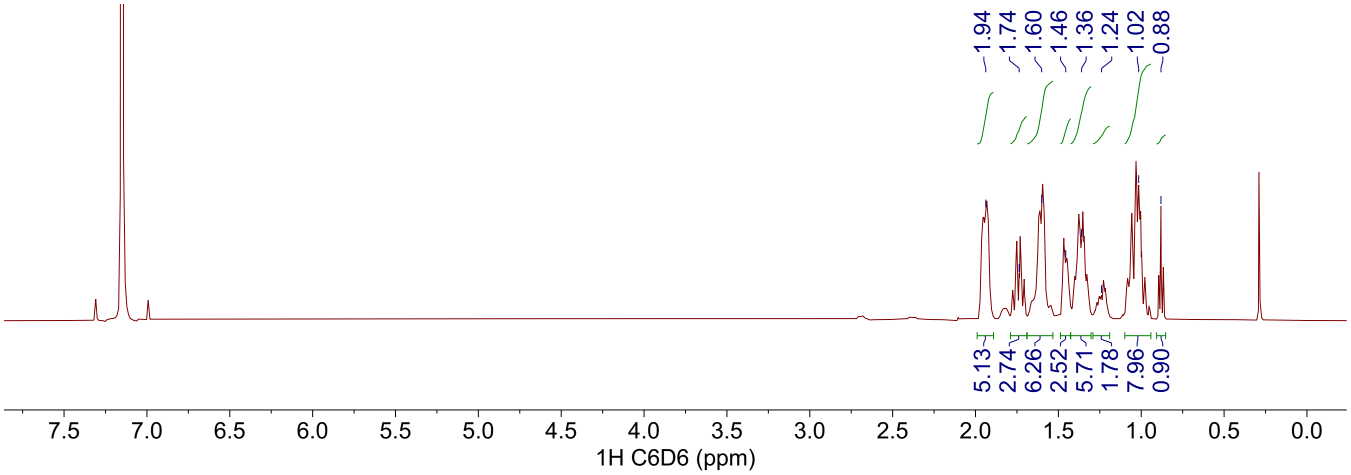

Figure S1. ^{1}H NMR spectrum of $^{125}TePCy_3$ in C_6D_6.

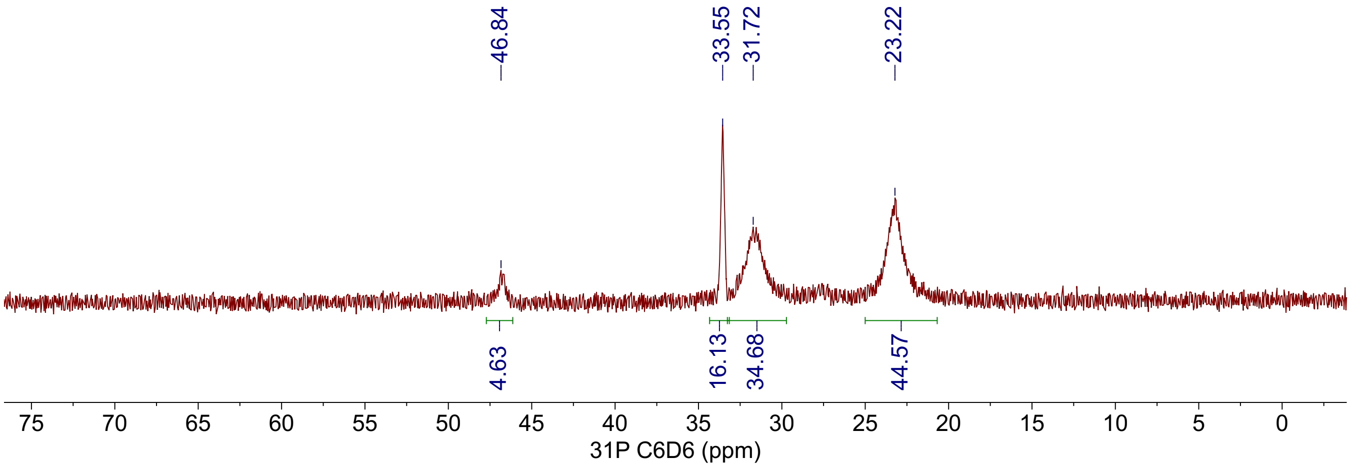

Figure S2. ^{31}P NMR of $^{125}TePCy_3$ in C_6D_6.

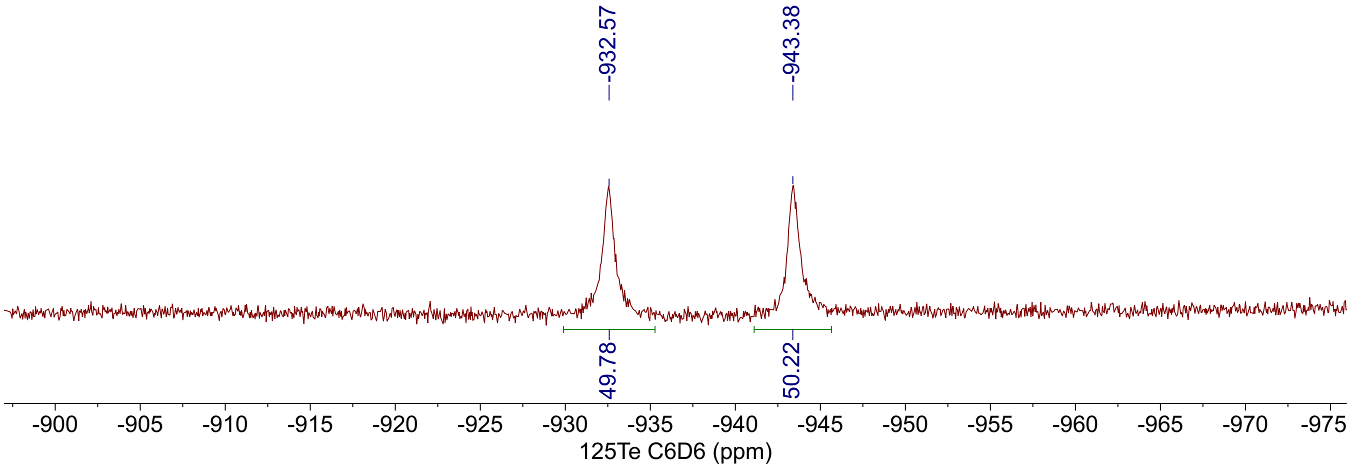

Figure S3. ^{125}Te NMR of $^{125}TePCy_3$ in C_6D_6.

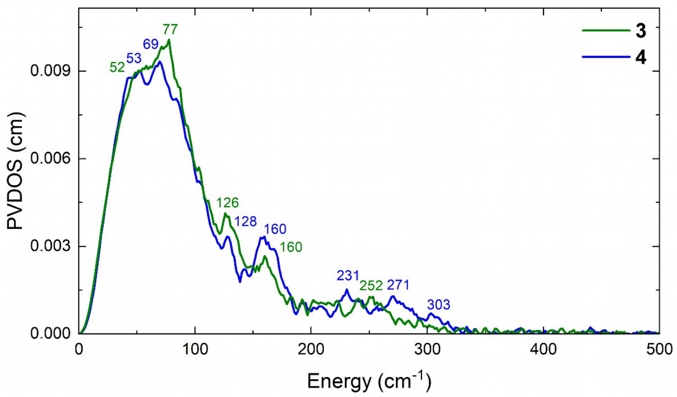

Figure S4. Comparison of experimental ^{125}Te NRVS spectra of complexes **3** and **4**.

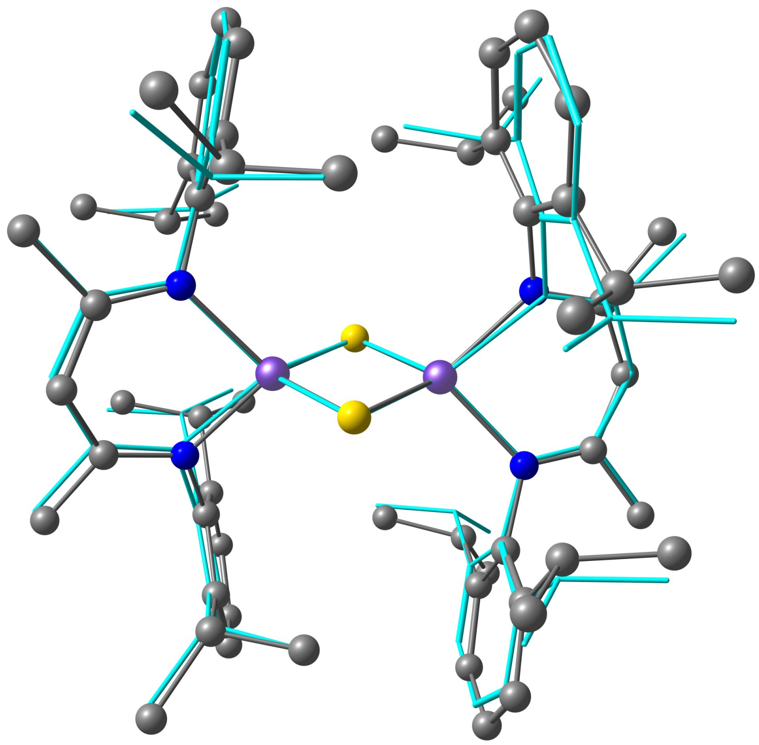

Figure S5. Comparison of DFT optimized (ball and stick) and experimental (wire) structure of complex **1**.

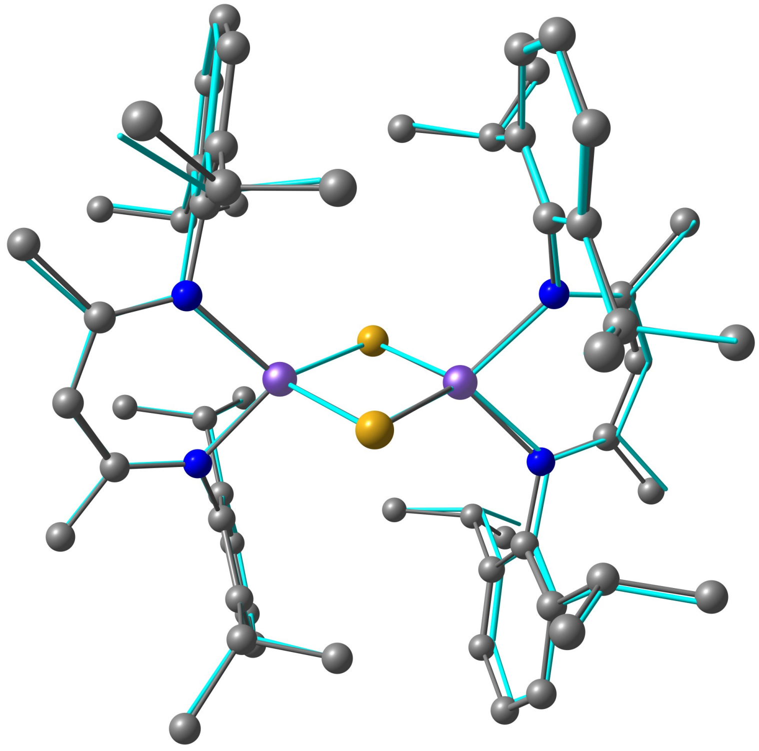

Figure S6. Comparison of DFT optimized (ball and stick) and experimental (wire) structure of complex **2**.

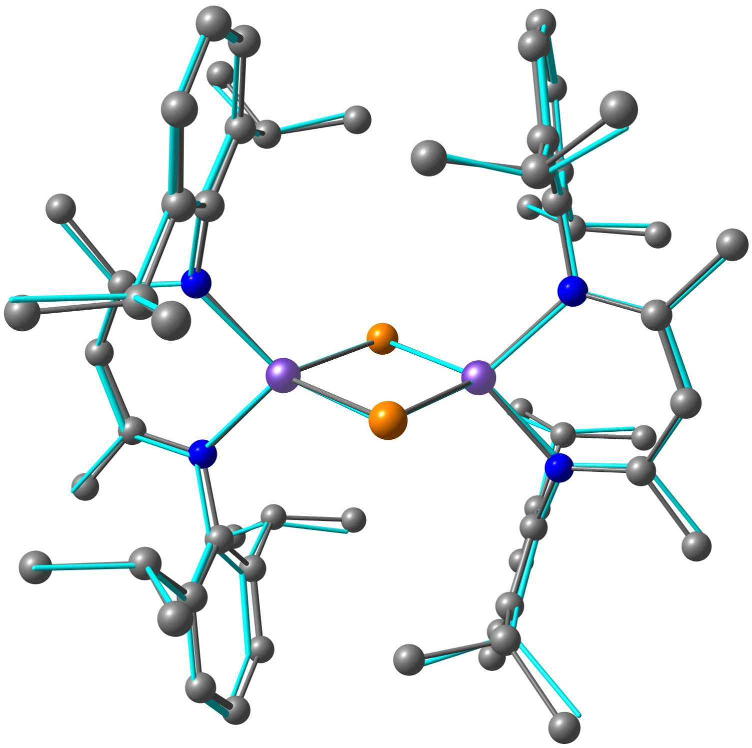

Figure S7. Comparison of DFT optimized (ball and stick) and experimental (wire) structure of complex **3**.

Table S1. Comparison of selected structural parameters of the experimental crystal structure and DFT optimized structures with different functionals

	experimental	bp86	b3lyp	pbe0	pbe	tpssh
**1**						
Fe – Fe (Å)	2.807	2.718	2.970	2.956	2.749	2.852
Fe – S_{avg} (Å)	2.238	2.202	2.293	2.283	2.209	2.251
Fe – S – Fe_{avg} (°)	77.7	76.2	80.7	80.6	77.0	78.6
**2**						
Fe – Fe (Å)	2.966	2.908	3.086	3.071	2.952	3.001
Fe – Se_{avg} (Å)	2.364	2.355	2.418	2.409	2.366	2.387
Fe – Se – Fe_{avg} (°)	77.7	76.2	79.2	79.1	77.2	77.89
**3**						
Fe – Fe (Å)	3.160	3.125	3.295	3.289	3.185	3.210
Fe – Te_{avg} (Å)	2.572	2.561	2.623	2.617	2.578	2.592
Fe – Te – Fe_{avg} (°)	75.7	75.2	77.8	77.8	76.3	76.5

Table S2. Calculated Mulliken spin populations for complexes **1** – **3** (Q=S, Se, Te) for broken-symmetry (BS) and high-spin (HS) solutions and relative energies.

		Fe1	Fe2	Fe_{total}	Q1	Q2	Q_{total}	Energy (kcal/mol)
**BS**	**1**	3.512	-3.083	0.429	0.245	0.213	0.458	0
	**2**	3.549	-3.272	0.277	0.262	0.344	0.606	0
	**3**	3.468	-3.271	0.197	0.307	0.383	0.690	0
**HS**	**1**	3.650	3.650	7.300	0.524	0.524	1.048	10.4
	**2**	3.606	3.606	7.212	0.585	0.585	1.170	6.4
	**3**	3.576	3.576	7.152	0.598	0.598	1.196	5.5

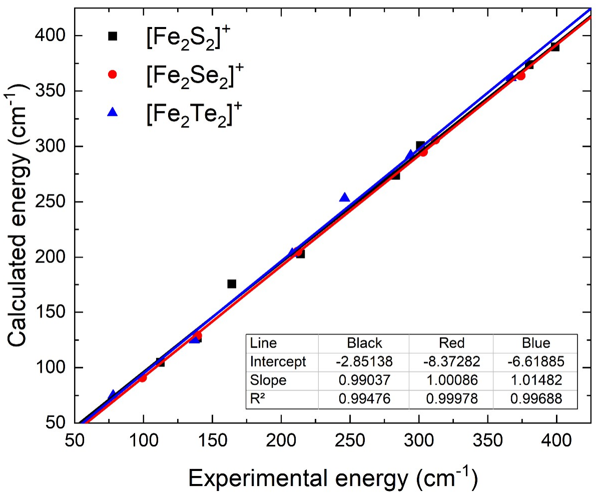

Figure S8. Comparison of energies of major spectral features in experimental and calculated NRVS spectra.

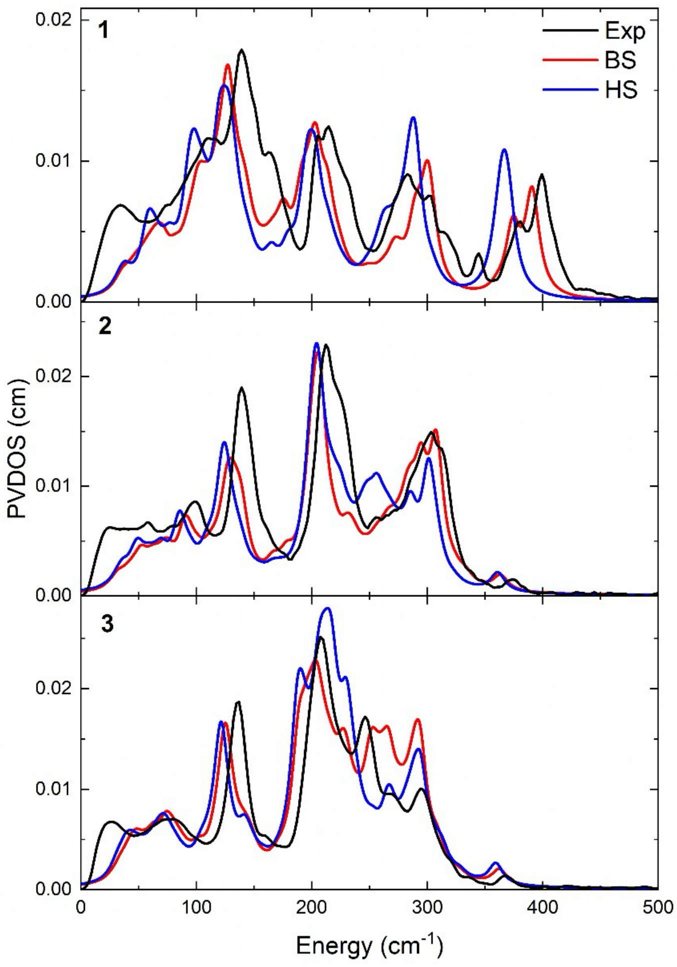

Figure S9. Comparison of broken spin (S = 1/2, red line) and high spin (S = 9/2, blue line) DFT calculated ^{57}Fe NRVS spectra with experimental spectrum (black line).

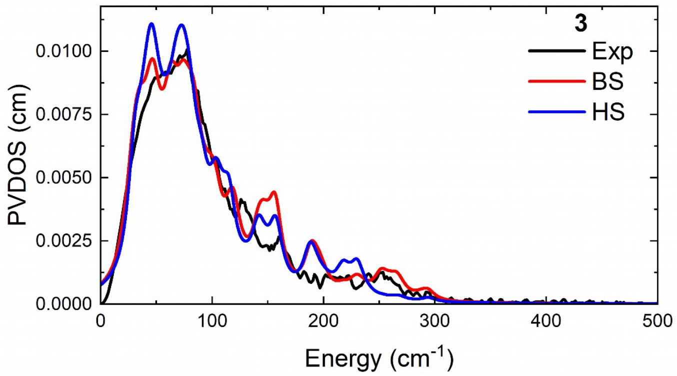

Figure S10. Comparison of broken spin (S = 1/2, red line) and high spin (S = 9/2, blue line) DFT calculated ^{125}Te NRVS spectra with experimental spectrum (black line) of complex **3**.

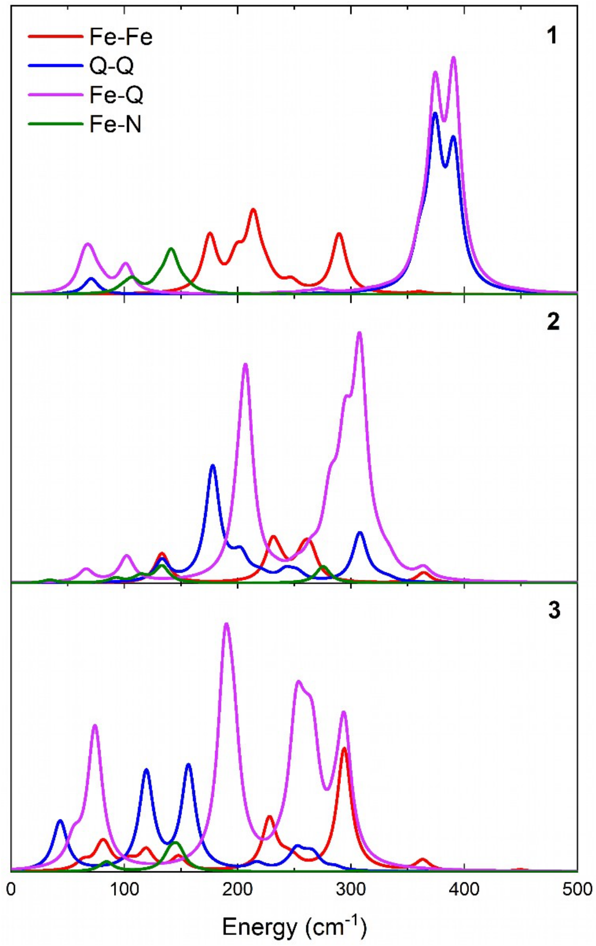

Figure S11. Kinetic energy distribution (KED) spectra of complexes **1** – **3**.

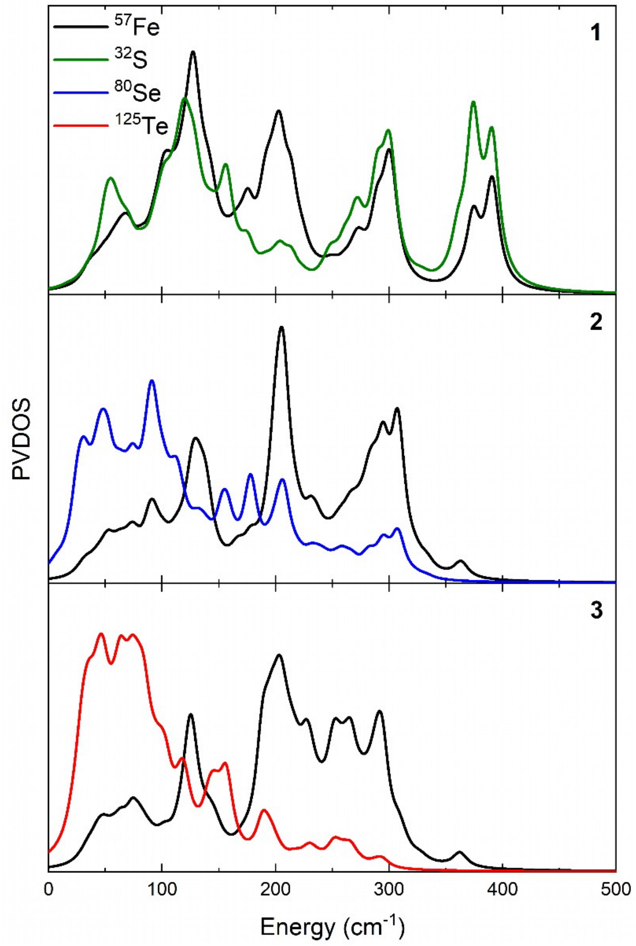

Figure S12. Comparison of calculated ^{57}Fe and Q (^{32}S, ^{80}Se, ^{125}Te) PVDOS spectra for complexes **1** – **3**.

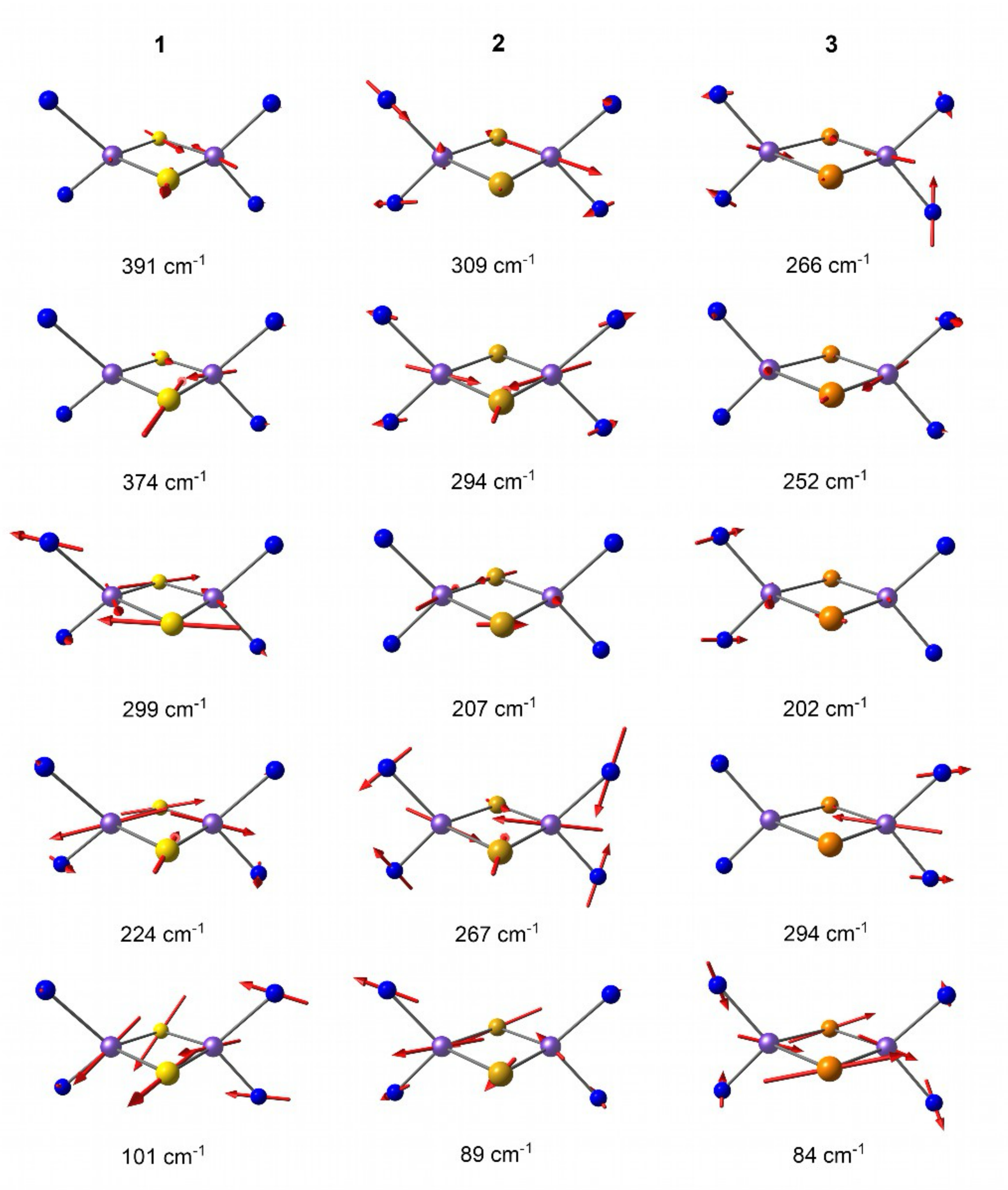

Figure S13. Representations of selected calculated normal modes of complexes **1** – **3**, which show significant energy change with changing chalcogen. Due to the clarity only $[Fe_2Q_2]^+$ core and coordinating N atoms are shown.

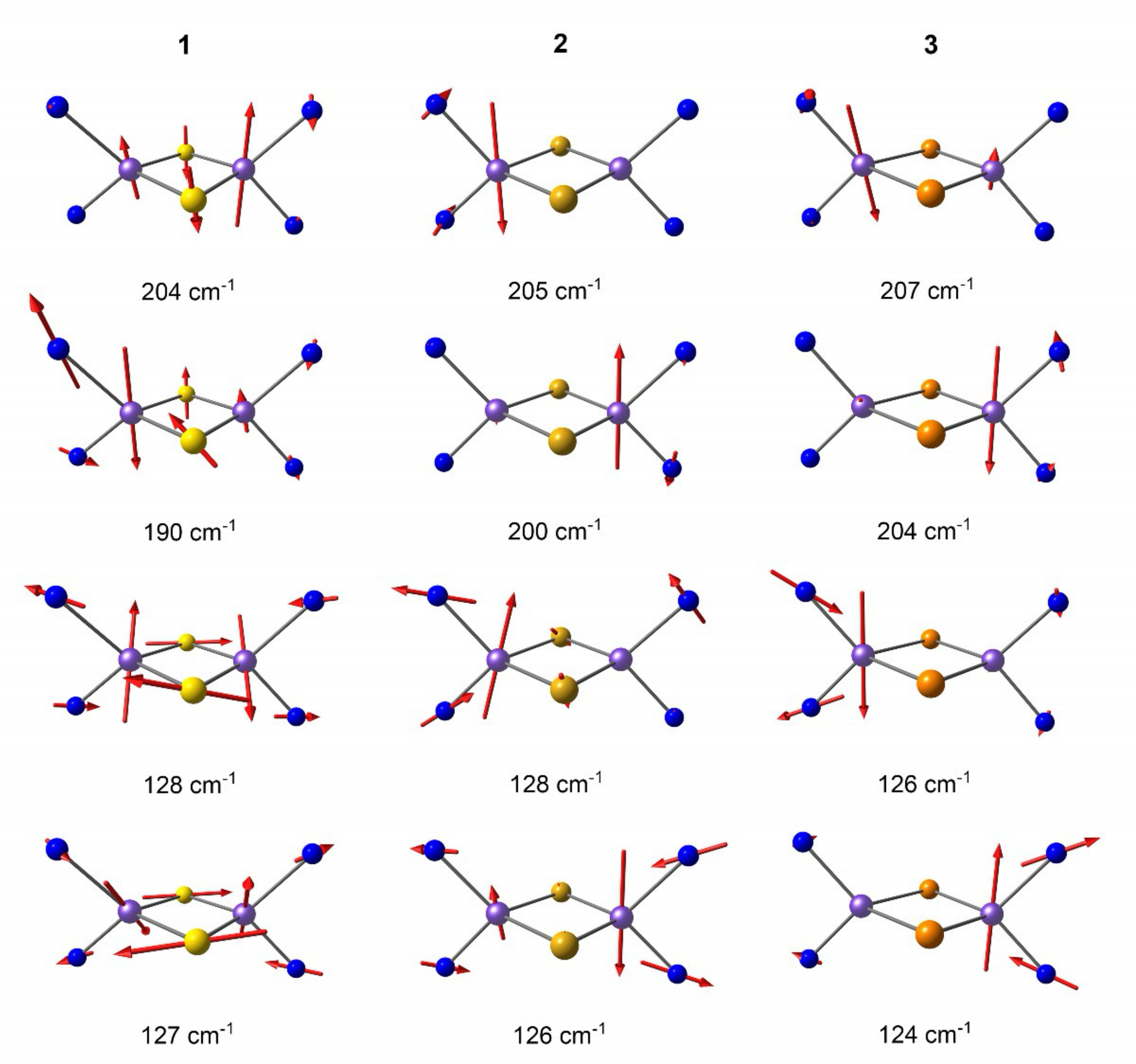

Figure S14. Representations of selected calculated normal modes of complexes **1** – **3**, which remain at similar energy with changing chalcogen. Due to the clarity only $[Fe_2Q_2]^+$ core and coordinating N atoms are shown.

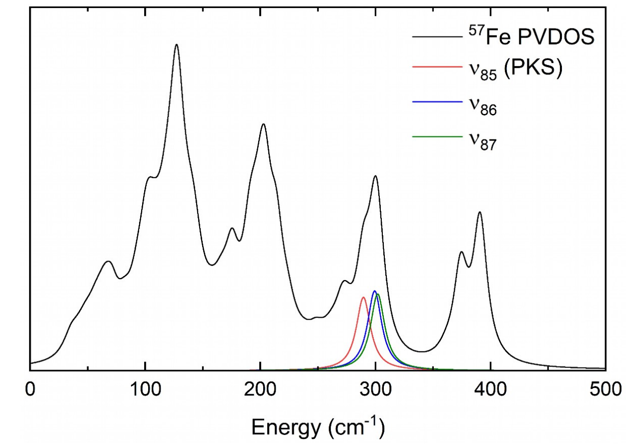

Figure S15. Representation of contributions from PKS and surrounding vibrations to total PVDOS spectrum of complex **1**.

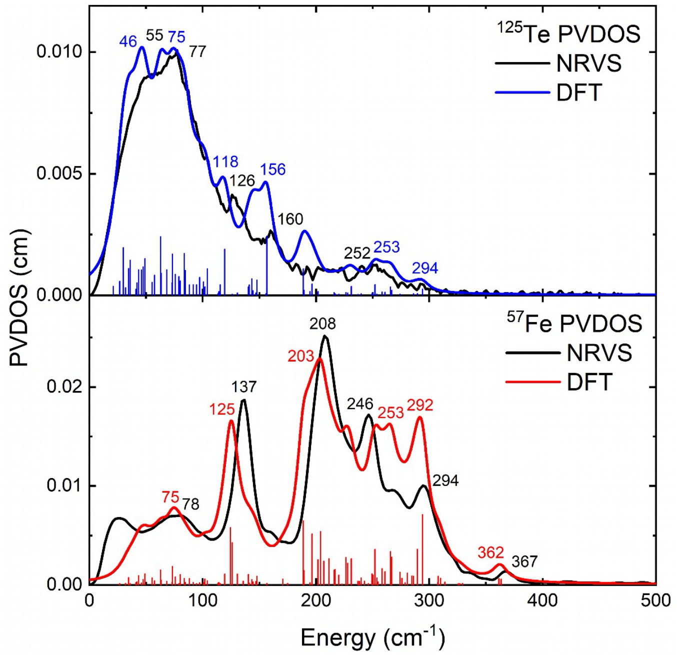

Figure S16. Comparison of ^{125}Te (top) and ^{57}Fe (bottom) NRVS spectra of complex **3**. Experimental spectra are depicted by black line, calculated spectra by blue and red lines, while individual transitions are depicted by blue and red vertical lines.

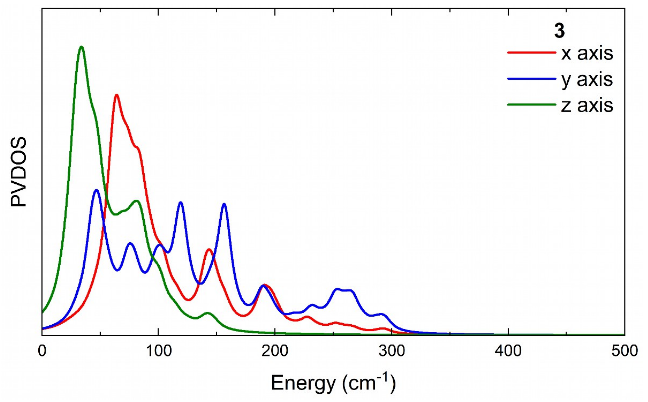

Figure S17. ^{125}Te mode compositions factors along x, y and z axes of complex **3**. X axis is defined along Fe-Fe bond, while the $[Fe_2Te_2]^+$ core is located in xy plane.

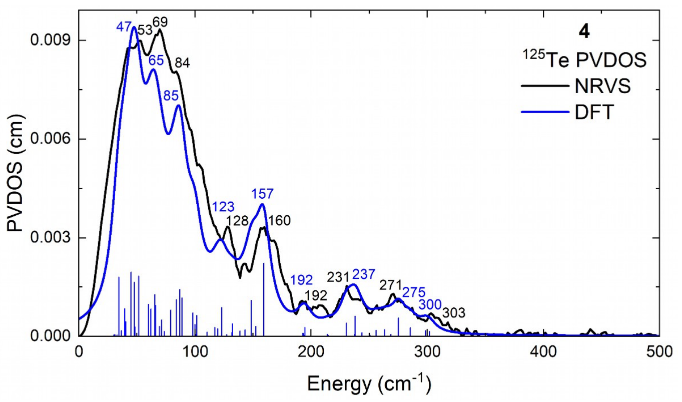

Figure S18. ^{125}Te NRVS spectrum of complex **4.** Experimental spectrum is depicted by black line, calculated spectrum by blue line, while individual transitions are depicted by blue vertical lines.

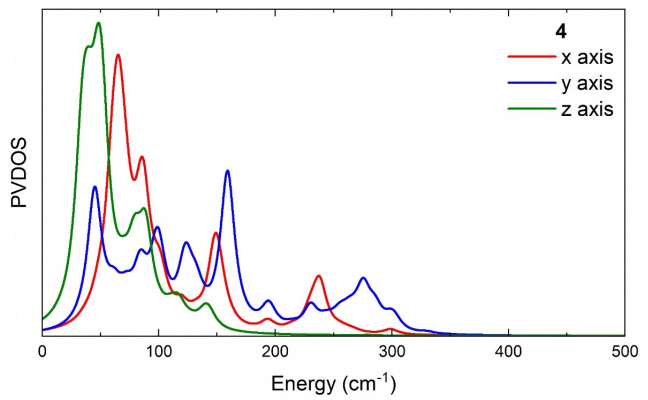

Figure S19. ^{125}Te mode compositions factors along x, y and z axes of complex **4**. X axis is defined along Fe-Fe bond, while the $[Fe_2Te_2]^{2+}$ core is located in xy plane.

Additional discussion of the ^{125}Te NRVS of **4**.

Transitions below 50 cm^{-1} are dominated by out-of-plane Te vibrations, while transitions over 100 cm^{-1} are mostly in-plane Te normal modes. Additionally, KED analysis (Figure S20) shows that sharp, moderately intense transitions at 128 and 160 cm^{-1} (in experimental spectrum) have significant Te-Te character. Analysis of individual normal modes revealed that transitions at 128 and 160 cm^{-1} correspond to the Te in plane breathing mode and Te-Te stretching mode, same as for complex **3** (123 and 159 cm^{-1}, respectively, Figure S21). This directly shows that changes in Fe oxidation states do not have significant influence on predominantly Te based vibrational modes. Due to the proximity to the Te-Te stretching mode, PKS vibration (at 148 cm^{-1}, calculated) is not well resolved in the experimental spectrum. The weak band at 303 cm^{-1}, which in comparison to spectrum **3** is more resolved, can be attributed to the Fe-Fe stretching vibration, based on KED and individual mode analysis (302 cm^{-1}, Figure S21). Two bands in experimental spectrum at 231 cm^{-1} and 271 cm^{-1} can be attributed to the $[Fe_2Q_2]^{2+}$ core in-plane normal mode (238 cm^{-1}, Figure S21) and out-of-phase breathing mode (275 cm^{-1}, Figure S21). These two bands overlap with transition corresponding to the Fe-Te stretching vibration (263 cm^{-1}, Figure S21), which prevents direct observation of Fe-Te stretching normal mode in experimental spectrum.

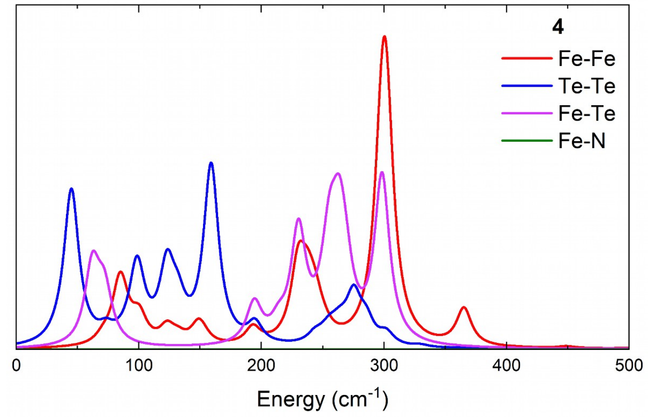

Figure S20. Kinetic energy distribution (KED) spectra of complex **4**.

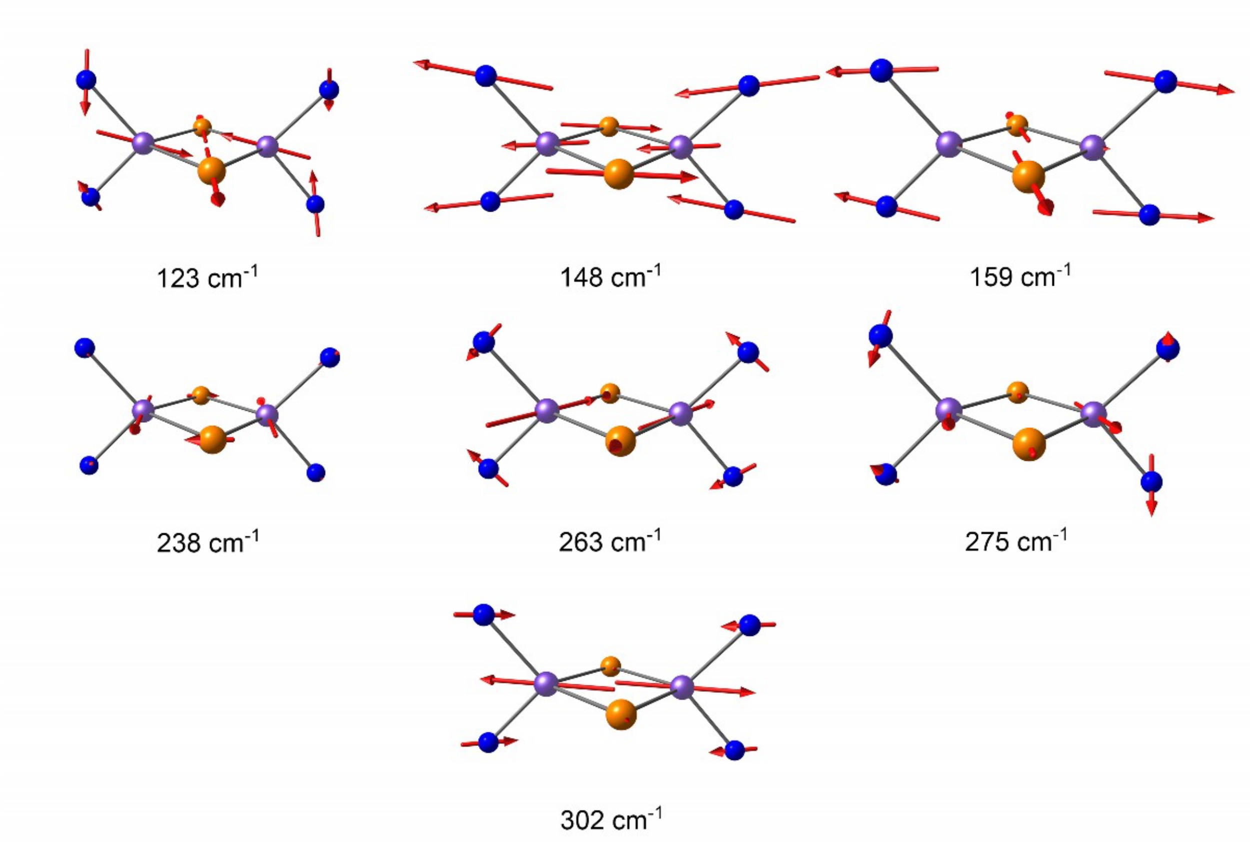

Figure S21. Representations of selected calculated normal modes of complex **4**. Due to the clarity only $[Fe_2Q_2]^{2+}$ core and coordinating N atoms are shown.

Table S3. Comparison of parameters *J*, *B* and vibronic coupling for complexes **1** – **3**.

	**1**	**2**	**3**
J (cm^{-1})[1]	-55	-50	-200
B (cm^{-1})[1]	110	165	750
ν_- (cm^{-1})	290	158	141
λ^2/k_- (cm^{-1})	2300	610	540

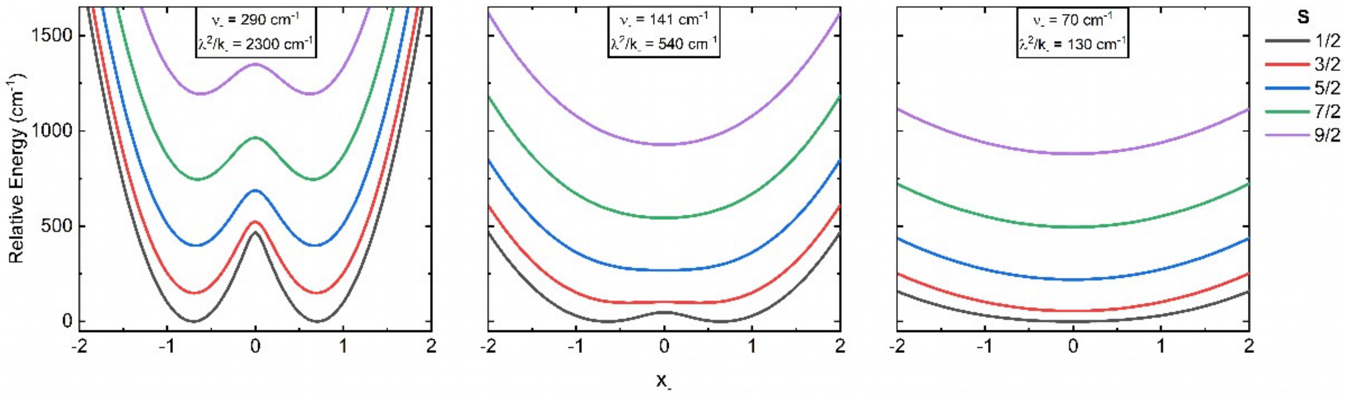

Figure S22. Ground and excited spin states in the PKS coordinate for the complex **1**, for different amounts of vibronic coupling.

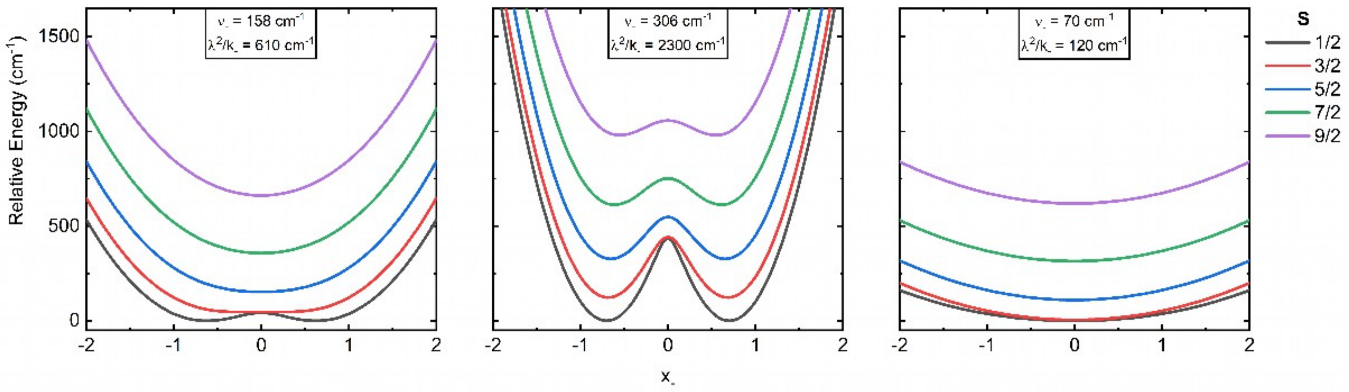

Figure S23. Ground and excited spin states in the PKS coordinate for the complex **2**, for different amounts of vibronic coupling.

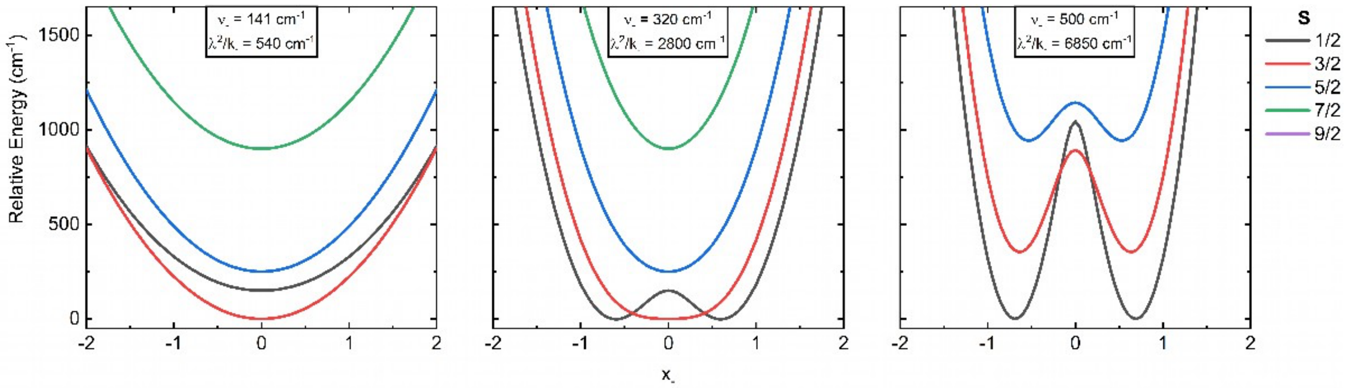

Figure S24. Ground and excited spin states in the PKS coordinate for the complex **3**, for different amounts of vibronic coupling.

References

1. Henthorn, J. T., Cutsail, G. E., Weyhermüller, T. & DeBeer, S. Stabilization of intermediate spin states in mixed-valent diiron dichalcogenide complexes. *Nat. Chem.* **14**, 328-333 (2022).
